# Preparation and Reliability and Validity Test of the Questionnaire on the Maintenance of Intravenous Catheter in ICU Nurses' Center

**DOI:** 10.1002/nop2.70145

**Published:** 2025-02-03

**Authors:** Feng Cheng, Hong Fu, Wei Yang

**Affiliations:** ^1^ Department of Critical Care Medicine First Affiliated Hospital of Anhui Medical University Hefei Anhui China

## Abstract

**Aim:**

To compile a questionnaire Knowledge, Attitude and Practices (KAP) of ICU nurses on central venous catheter maintenance and test the reliability and validity of the questionnaire.

**Design:**

Compile a questionnaire, conduct a questionnaire survey and test the reliability and validity of the questionnaire.

**Methods:**

The initial questionnaire was formed based on the theory of KAP, combined with literature review and Delphi expert correspondence method; a questionnaire survey was conducted among 334 ICU nurses in five tertiary hospitals, and the reliability and validity of the questionnaire were tested and a formal questionnaire was compiled.

**Results:**

The ICU nurses' CVC maintenance KAP questionnaire included 31 items in three dimensions of knowledge, attitude and behaviour. The item content validity (I‐CVI) and questionnaire content validity (S‐CVI) were 0.889 ~ 1.000 and 0.974, respectively. Exploratory factor analysis extracts three common factors (dimensions), the cumulative variance contribution rate is 65.656%, the Cronbach'*α* coefficient of the total questionnaire is 0.843, and the Cronbach's α coefficients of the three dimensions of KAP are 0.754, 0.887 and 0.940, respectively. The split‐half reliability is 0.816. The test–retest reliability of the total questionnaire is 0.813.

**Conclusion:**

The ICU nurses' CVC maintenance KAP questionnaire has good reliability and validity, stable results, comprehensive coverage, and high feasibility. It can be used as a measurement tool to evaluate the maintenance level of CVC for ICU nurses in China.

**No Patient or Public Contribution:**

No patient or public contribution.

## 1 Introduction

A central venous catheter (CVC) is a large‐bore CVC placed through the skin using sterile techniques in certain clinical situations. In adult patients, the three placement sites for CVCs are the internal jugular vein, femoral vein, and subclavian vein, with the catheter reaching the right atrium via the puncture site (Bleichmder 1912). CVCs have been widely used in the clinical treatment of critically ill patients and are an important route for monitoring the condition of critically ill patients, infusing fluids and blood, providing total parenteral nutrition and administering life‐saving drugs (Lafuente Cabrero et al. 2023). However, due to the fact that the patients' conditions are generally severe, invasive procedures are often performed, and their immune function is low, deep venous catheterization is associated with an increasing number of complications, including catheter‐related bloodstream infections, catheter occlusion, slippage, pneumothorax and air embolism (Wang, Sun, and Wang 2019). Of these, improper maintenance is the main cause of complications.

Nurses monitor CVCs to prevent complications such as infection, pneumothorax, hematoma, bleeding or extravasation, so that corrective measures can be taken in a timely manner to improve medical care (Sun et al. 2020). The knowledge, attitude, and practices (KAP) of CVC care among intensive care unit (ICU) nurses have a significant impact on the occurrence of CVC complications. To date, no specific questionnaires have been found to assess the KAP of ICU nurses regarding CVC maintenance, making it difficult to standardise and improve the regulation of CVC maintenance by ICU nurses. Therefore, this study developed an ICU Nurse Central Venous Catheter Maintenance Knowledge‐Attitude‐Practice Questionnaire to provide a reliable theoretical basis for evaluating ICU nurses' knowledge, attitudes and behaviours towards CVC maintenance.

## 2 Materials and Methods

### 2.1 Research Object

#### 2.1.1 Consulting Experts by Mail

Medical experts and psychological experts with intermediate technical titles and above engaged in critical care, clinical medicine, nursing management for more than 10 years; (1) Bachelor degree or above; (2) Interested and willing to participate in the study. The study selected 19 experts from east, west and central China.

#### 2.1.2 Pre‐Survey Subjects

In July 2020, 20 ICU nurses from REDACTED were selected by convenience sampling to conduct a pre‐survey on questionnaires. The eligibility criteria are: (1) Have a nurse practicing qualification certificate issued by the Ministry of Health within the valid registration period; (2) Years of front‐line nursing work in ICU > 1 year; (3) Able to correctly understand the questionnaire content; (4) Informed consent and voluntary participation in this survey. Exclusion criteria: training, rotation, practice nurses; Nurses who are out of work due to marriage, illness, childbirth, etc.

#### 2.1.3 Formal Survey

Subjects were selected by convenience sampling method. From July to September 2020, on‐site questionnaire survey was conducted among ICU nurses in REDACTED. The inclusion and exclusion criteria are the same.

### 2.2 Research Methods

#### 2.2.1 Preliminary Questionnaire Construction

A multidisciplinary team consisting of two critical care clinical experts, one critical care research expert, one intravenous therapy expert, one psychological measurement expert, one statistician and three graduate students was established. Based on the theory of knowledge, belief and action (Li and Liu 2012) and referring to domestic and foreign research and related guidelines (Gorski et al. 2016; Chinese Nursing Association intravenous Infusion Therapy Professional Committee 2019; Estrada‐Orozco et al. 2020; National Health and Family Planning Commission 2014) as well as expert group discussions, the initial questionnaire compiled included 14 items in the knowledge dimension, 8 items in the attitude dimension, and 26 items in the behaviour dimension. Using the Delphi method, two rounds of expert correspondence were conducted from July to September 2020. According to the concentration degree of expert opinions, coordination degree of expert opinions and other indicators, combined with expert suggestions, the initial items of the questionnaire were screened and modified to form a pre‐test version of the questionnaire. The questionnaire contains 55 items in total, including 16 items in the knowledge dimension, 9 items in the attitude dimension and 30 items in the behaviour dimension. Five points are scored for all correct answers to knowledge items, 1 point is scored for all correct answers to multiple choice items, and 0 points is scored for all wrong answers. All attitude and behaviour items are scored on the Likert five‐point scoring scale (Likert 1932): strongly agree, agree, not necessarily, disagree and strongly disagree five kinds of answers, which are recorded as 5, 4, 3, 2 and 1 points respectively.

#### 2.2.2 Pre‐Survey

The pre‐test version of questionnaire was used to conduct a questionnaire survey on 20 nurses who met the inclusion criteria. The questionnaire was collected on site with an effective recovery rate of 100%. After the preliminary survey, no nurses put forward new demands, and the questionnaire content was not modified. So the initial version of the questionnaire consisted of three dimensions and 55 items for clinical testing.

#### 2.2.3 Formal Investigation

An on‐site questionnaire survey was conducted on 360 nurses who met the inclusion criteria using the self‐prepared general data questionnaire of nurses and the CVC maintenance questionnaire of ICU nurses (clinical test version), and 334 valid questionnaires were collected.

#### 2.2.4 Item Analysis

Items are screened based on data from formal surveys. The following two methods are mainly adopted in this study: (1) critical ratio (CR): also known as extreme value method, that is, the scores of all subjects are sorted from the largest to the smallest. If the score is in the top 27%, that is, the sample of the high group, while the score is in the bottom 27%, that is, the sample of the low group. Through the *t*‐test analysis of two independent samples, if the CR value of the item is > 3 or the difference is statistically significant (*p* < 0.05), the item will be retained; otherwise, it will be deleted (Xiaoyong 2020). (2) Total correlation analysis: The correlation coefficient between each item and the total score was calculated. If the correlation coefficient was not significant (*p* > 0.05) or *r* < 0.4, the item was deleted (Xiaoyong 2020). Based on the project analysis results, a questionnaire on the maintenance of CVC for ICU nurses was formed (preliminary version).

#### 2.2.5 Reliability and Validity Analysis

The content validity of this study was obtained by letter consultation with experts. The validity of questionnaire structure was tested by exploratory factor analysis. The reliability is tested by internal consistency reliability and retest reliability.

### 2.3 Quality Control

After unified training, the quality control investigators introduced the research purpose and precautions to the nurses with unified guidance. After completing the questionnaire, they checked the filling status on the spot and corrected and supplemented the errors and omissions in time.

### 2.4 Ethical Principles

This study follows the principles of voluntariness and confidentiality to avoid any harm to the participants. The study was reviewed and approved by the Ethics Committee of REDACTED (Approval number: REDACTED).

### 2.5 Statistical Methods

Excel 2013 was used for data entry and sorting. SPSS 26.0 and Amos24.0 were used for statistical analysis of the data. The measurement data were described by mean ± standard deviation, and the counting data were described by frequency and percentage. The difference was statistically significant when *p* < 0.05. The correlation between items and total score and the critical ratio method were used to screen and analyse the questionnaire items (Xiaoyong 2020). Factor analysis was used to test the validity of the questionnaire. The validity analysis included content validity and structure validity. Expert evaluation method was used for Content Validity. Item of Content Validity Index (I‐CVI): For each item, the number of experts with a rating of 3 or 4 divided by the total number of experts participating in the evaluation is the I‐CVI. Scale of Content Validity Index (S‐CVI): The number of times of 3 or 4 ratings divided by the number of evaluations. Exploratory factor analysis and confirmatory factor analysis were used for structural validity. Cronbach α coefficient and broken half reliability were used to describe the validity of the questionnaire. The validity of item level content and item level content were calculated by using expert evaluation method. Exploratory factor analysis was used to test the validity of the structure. If KMO ≥ 0.8, *p* < 0.05 indicates that exploratory factor analysis is suitable. Principal component analysis is used to extract common factors with eigenvalues > 1, and maximum variance orthogonal spin method is used to delete items with factor loads < 0.4 (Zhang and Dong 2018). Confirmatory factor analysis was used to test the fit degree of each dimension and item of the questionnaire. Cronbach's *α* and broken half reliability were used to evaluate the reliability of the questionnaire (Wu 2003).

## 3 Results

### 3.1 Expert Correspondence

Among the 19 experts who completed 2 rounds of Delphi expert correspondence, 4 were male and 15 were female; Age < 40 years old 5 people, 40 ~ 50 years old 11 people, > 50 years old 3 people; 10 students have bachelor degree, 5 master degree and 4 doctor degree. Professional titles are intermediate 6 people, associate senior 10 people, senior 3 people; The working years are 10 ~ 20 years 8 people, 21 ~ 30 years 9 people, 30 years or more 2 people; Specialist expertise includes critical care (7 people), intravenous care (4 people), nursing management (4 people), nursing research (2 people), clinical medicine (1 person), psychology (1 person).

With the average score of importance < 3.50 and coefficient of variation > 0.25 as the deletion criteria (Xiaoyong 2020), entries were added, deleted and modified based on expert opinions. The recovery rates of the two rounds of expert correspondence questionnaires were 95.0% (19/20) and 100% (19/19), and the positive coefficient of experts were 95.00% and 100.00%, respectively. The Cr of the two rounds of expert consultation is 0.83 ~ 0.98, which can be considered that the experts who participated in this letter have high authority. After the first round of consultation, 12 experts made constructive written suggestions, and after the second round, 4 experts made written suggestions. The revised questionnaire developed after the first round of consultation modified the expression of 6 items, adding 2 items of knowledge dimension, 2 items of attitude dimension and 4 items of behaviour dimension. After the second round of consultation, one item in the attitude dimension was deleted, and the expression ways of the two items were further modified. The consensus questionnaire formed after the letter consultation contained 55 items. The Kendall coordination coefficient of the two rounds was statistically significant (*χ* ^2^ test, *p* < 0.001, Table 1), indicating a good degree of coordination of expert opinions, so the conclusion is credible.

**TABLE 1:** Kendall's coordination coefficient (Kendall's W) in correspondence with Delphi experts.

Number of correspondence rounds	Item	Item	Number of experts	Kendall's W	*χ* ^2^	*df*	*p*
First‐round	Knowledge	14	19	0.249	61.503	13	0.000
	Attitude	8	19	0.163	21.64	7	0.003
	Behaviour	26	19	0.138	65.719	25	0.000
	Entirety	48	19	0.200	178.356	47	0.000
Second round	Knowledge	16	19	0.104	33.696	15	0.009
	Attitude	10	19	0.553	94.5	9	0.000
	Behaviour	30	19	0.118	65.201	29	0.000
	Entirety	56	19	0.280	303.102	55	0.000

### 3.2 Project Analysis

#### 3.2.1 Correlation Coefficient Method

Pearson correlation coefficient method was used to analyse the correlation between each item and the total score of the questionnaire. Item 2 item 3, 6, 8, 9, 11, 12, 13, 14, 15 in addition to knowledge, attitude, behaviour items 1, 2, 8, 11, 16, 18, 19, 20, 22, 24, 25, 27, 28, 29 (*p* > 0.05), the rest of the entries correlation coefficient of 0.40 or higher.

#### 3.2.2 The Critical Ratio Method

Conducted independent sample *t* test to analyse the critical difference between the items in the high and low groups. The decision values of all items in the questionnaire ranged from 3.241 to 11.582 with statistical significance (*p* < 0.001), and the results showed that no items were deleted, as shown in Table 2.

**TABLE 2:** 95% confidence intervals for the decision values and differences of the entries.

Item	Decision value	95% confidence interval for the difference		Item	Decision value	95% confidence interval for the difference	
		Lower limit	Upper limit			Lower limit	Upper limit
Knowledge1	9.282*	1.674	2.582	Behavior3	7.976*	0.493	0.817
Knowledge2	11.582*	2.377	3.355	Behavior4	7.936*	0.354	0.590
Knowledge4	3.439*	0.388	1.439	Behavior5	9.075*	0.515	0.802
Knowledge5	6.563*	1.002	1.869	Behavior6	6.652*	0.343	0.633
Knowledge7	3.904	0.840	2.563	Behavior7	8.023*	0.372	0.616
Knowledge10	3.241*	0.496	2.045	Behavior9	5.635*	0.285	0.594
Knowledge16	11.072*	1.367	3.315	Behavior10	7.519*	0.382	0.655
Attitude1	3.748*	0.080	0.260	Behavior12	7.935*	0.424	0.706
Attitude3	4.940*	0.202	0.473	Behavior13	8.112*	0.402	0.661
Attitude4	7.713*	0.428	0.722	Behavior14	7.383*	0.365	0.634
Attitude5	5.806*	0.314	0.639	Behavior15	6.221*	0.391	0.757
Attitude6	5.497*	0.286	0.607	Behavior17	6.762*	0.346	0.631
Attitude7	7.504*	0.428	0.735	Behavior21	5.661*	0.337	0.700
Attitude8	6.199*	0.363	0.703	Behavior23	6.189*	0.277	0.538
Attitude9	5.867*	0.355	0.717	Behavior26	7.303*	0.471	0.820
				Behavior30	5.673*	0.329	0.680

*Note:* The number in the entry column is the entry number.

* means *P < *0.001.

### 3.3 Reliability and Validity Test

#### 3.3.1 Validity Analysis

##### 3.3.1.1 Content Validity

The results of evaluation by nine experts showed that CVI (I‐CVI) = 0.889 ~ 1.000, CVI(S‐CVI) = 0.974, and the content validity was good.

##### 3.3.1.2 Structural Validity

**FIGURE 1:**
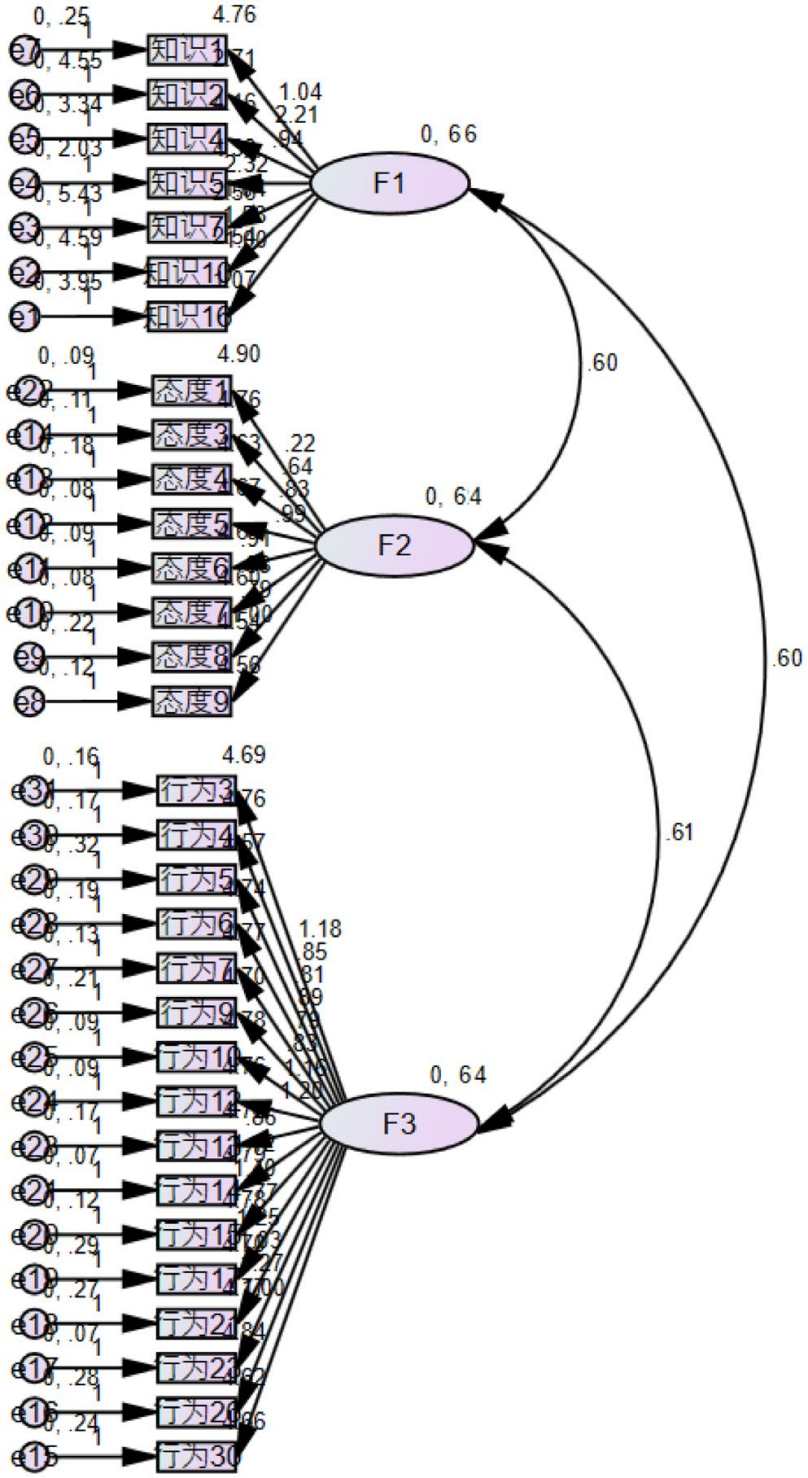
Standardised three‐factor structural equation model (*n* = 184). Path coefficient, F1: knowledge dimension of ICU nurses' CVC maintenance, F2: attitude dimension of ICU nurses' CVC maintenance, F3: behaviour dimension of ICU nurses' CVC maintenance.

Based on the formal investigation data, factor analysis was performed, and the KMO value was 0.913, and Bartlett sphericity test reached a significant level (c2 = 5886.897, *p* < 0.001), which was suitable for exploratory factor analysis. In this paper, 31 items were analysed by principal component analysis and variance maximum rotation method. There were three common factors (F1–F3) with feature root *λ* > 1, and the corresponding total interpretation rate of variation was 65.656%, indicating that the questionnaire had good structural validity. The factor load and commonality of each item of the questionnaire are shown in Table 3. The fit index of the two‐factor structural equation model is shown in Table 4. The standardised path analysis is shown in Figure 1.

**TABLE 3:** Factor load and common degree of each item after rotation (*n* = 184).

Item	F1 (Knowledge = 7)	F2 (Attitude = 8)	F3 (Behaviour = 16)	Communality
Knowledge16	0.850	—	—	0.768
Knowledge4	0.792	—	—	0.655
Knowledge7	0.602	—	—	0.632
Knowledge10	0.694	—	—	0.442
Knowledge2	0.739	—	—	0.584
Knowledge5	0.647	—	—	0.582
Knowledge1	0.541	—	—	0.520
Attitude7	—	0.847	—	0.809
Attitude1	—	0.802	—	0.638
Attitude6	—	0.803	—	0.757
Attitude8	—	0.784	—	0.686
Attitude5	—	0.764	—	0.818
Attitude9	—	0.764	—	0.752
Attitude4	—	0.734	—	0.681
Attitude3	—	0.618	—	0.596
Behavior6	—	—	0.795	0.767
Behavior5	—	—	0.775	0.685
Behavior26	—	—	0.742	0.629
Behavior30	—	—	0.729	0.583
Behavior4	—	—	0.704	0.691
Behavior17	—	—	0.614	0.545
Behavior23	—	—	0.659	0.767
Behavior3	—	—	0.625	0.709
Behavior9	—	—	0.572	0.560
Behavior15	—	—	0.714	0.762
Behavior13	—	—	0.622	0.598
Behavior7	—	—	0.603	0.553
Behavior10	—	—	0.676	0.697
Behavior21	—	—	0.654	0.664
Behavior14	—	—	0.688	0.737
Behavior12	—	—	0.659	0.679

*Note:* “‐” indicates that the absolute value of the load in this article is less than or equal to 0.400. The entry column number is the entry number.

**TABLE 4:** Confirmatory factor analysis (*n* = 184).

Statistical test	Fit the standard or critical value	Test result data model	Fit judgement
CMIN/DF	1 ≤ *χ* ^2^ */v* ≤ 3	2.907	Yes
RMR	< 0.05	0.019	Yes
RMSEA	< 0.08	0.071	Yes
GFI	> 0.90	0.973	Yes
AGFI	> 0.90	0.928	Yes
TLI	> 0.90	0.943	Yes
IFI	> 0.90	0.979	Yes
CFI	> 0.90	0.977	Yes
PGFI	> 0.50	0.675	Yes

*Note:* v is the degree of freedom, and the model has 10^9^° of freedom.

#### 3.3.2 Reliability Analysis

The Cronbach's *α* coefficient of the questionnaire was 0.843, and the Cronbach's α coefficient of each dimension was 0.754, 0.887, 0.940. The partial half reliability was 0.816, and the retest reliability was 0.813. It shows that the questionnaire has reliability and stability.

## 4 Discussion

### 4.1 Significance of Questionnaire Preparation

The standard catheter maintenance performed by nurses can reduce complications, extend the service life of the catheter and reduce the economic burden of patients, which is of great significance in the whole course of intravenous therapy. In order to improve the quality of CVC maintenance, it is necessary to have a scientific and quantitative measurement tool to evaluate the knowledge and practice of ICU nurses on CVC maintenance, so as to formulate targeted interventions to improve the quality of CVC maintenance and reduce the occurrence of CVC complications. At present, there are no evaluation tools to evaluate the knowledge and practice of ICU nurses' central venous pipeline maintenance at home and abroad. The existing universal knowledge and practice assessment tools and the standard questionnaire for intravenous therapy involve a wide range of contents (Yao 2019; Chen et al. 2022), which is difficult to reflect the specific knowledge, attitude and behaviour of CVC maintenance. Specific assessment tools such as peripherally inserted central catheter and the questionnaire providing catheter maintenance knowledge and practice are highly targeted (Zhang et al. 2019; Ren et al. 2024) and are not applicable to the assessment of catheter maintenance knowledge and practice. In view of this, it is of practical significance to construct a questionnaire on the knowledge and practice of ICU nurses' central venous pipeline maintenance and provide a targeted quantitative evaluation tool for clinical practice.

### 4.2 The Questionnaire Is Applicable

In this study, knowledge, belief and practice were selected as the theoretical framework to construct the dimension of the questionnaire, and the questionnaire items were constructed in combination with relevant literature and expert consensus on Clinical venous catheter maintenance and Operation (Chinese Nursing Association intravenous Infusion Therapy Professional Committee 2019). After group discussion, the item pool was established by brainstorming method. Delphi expert correspondence method was used to select items and form the initial questionnaire. In this study, medical experts and psychological experts from intravenous nursing, critical care, clinical medicine and nursing management were selected, and the initial entries were modified and screened through two rounds of Delphi expert correspondence, and professional opinions on the questionnaire were put forward from multiple angles and directions. The data were collected by investigating ICU nurses, and the reliability of the questionnaire was analysed by various statistical methods. The higher the score of the questionnaire, the higher the knowledge of CVC maintenance, the better the attitude and the more normative the behaviour. This questionnaire met nine aspects of CVC maintenance, including evaluation, tube flushing, tube sealing, dressing replacement, catheter fixation, infusion joint, catheter removal, and infection prevention and quantitatively assessed the knowledge, belief and practice of ICU nurses on CVC maintenance. The language of the questionnaire is easy to understand, and it usually takes less than 10 min to fill in, which is easy to promote and apply.

### 4.3 The Questionnaire Has Good Reliability and Validity

The preparation process strictly follows the standardisation of questionnaire preparation. The questionnaire in this study has good stability. Principal component analysis and variance maximum rotation method showed that there were three common factors with eigenroot *λ* > 1, and the total interpretation rate of corresponding variation was 65.656%, indicating that the questionnaire structure was valid.

The quantitative questionnaire for the maintenance of CVC in ICU nurses has high reliability, validity and operability, and can be used as a quantitative evaluation tool for the maintenance of CVC in ICU nurses. However, this study only decided to delete items from the perspective of statistical analysis, without considering the integrity of catheter maintenance. In addition, the ICU nurses involved were all from Anhui province, which has a certain region, and the sample size and cross‐provincial multi‐center studies need to be increased in the future, so as to improve the questionnaire.

## Ethics Statement

This paper was approved by the Ethics Committee of the First Affiliated Hospital of Anhui Medical University (Approval number: Kuai‐Lun Review of the First Affiliated Hospital of An Medical University‐P2020‐17‐12), and we were in accordance with the 1975 Helsinki declaration and its later amendments.

## Consent

The authors have nothing to report.

## Conflicts of Interest

The authors declare no conflicts of interest.

## Data Availability

All data generated or analysed during this study are included in this published article.
